# Can Prebiotics Support Microbial Metabolic Function Following Low-FODMAP Restriction in Irritable Bowel Syndrome?

**DOI:** 10.3390/nu18213535

**Published:** 2026-10-27

**Authors:** Francesca Gorini, Alessandro Tonacci, Morena Gabriele, Andrea Borghini

**Affiliations:** 1 Institute of Clinical Physiology, National Research Council, Via Moruzzi 1, 56124 Pisa, Italy; alessandro.tonacci@cnr.it (A.T.); andrea.borghini@cnr.it (A.B.); 2 Institute of Agricultural Biology and Biotechnology, National Research Council, Via Moruzzi 1, 56124 Pisa, Italy; morena.gabriele@ibba.cnr.it

**Keywords:** irritable bowel syndrome, IBS, low-FODMAP diet, prebiotics, microbiota, personalized nutrition

## Abstract

Irritable bowel syndrome (IBS) is one of the most common disorders of gut–brain interaction, and dietary interventions play a central role in symptom management. Among these, the low-fermentable oligosaccharides, disaccharides, monosaccharides, and polyols (low-FODMAP) diet has been shown to improve abdominal pain, bloating, and altered bowel habits in a substantial proportion of patients. However, restriction of fermentable carbohydrates may reduce the availability of substrates for the gut microbiota and has been associated with reductions in selected microbial taxa, including *Bifidobacterium*, raising questions about its effects on microbial ecology and metabolism. Dietary prebiotics selectively stimulate gut microorganisms and promote microbial fermentation and the production of metabolites, including short-chain fatty acids, that may contribute to intestinal homeostasis. However, many prebiotics are also highly fermentable substrates, and their gastrointestinal effects may vary substantially among individuals with IBS. This creates a challenge in balancing symptom control with continued exposure to substrates that may support microbial and metabolic functions. In this narrative review, we critically examine the metabolic and clinical implications of dietary prebiotics in IBS, with particular emphasis on their potential role following low-FODMAP restriction. We discuss their effects on gut microbiota composition and function, microbial metabolites, and clinically relevant outcomes, with particular attention to the relationship between microbial and symptomatic responses. We further consider the potential role of structured reintroduction and personalization of tolerated fermentable substrates in maintaining dietary variety and microbial substrate availability while minimizing gastrointestinal symptoms. Finally, we discuss interindividual variability in responses to fermentable substrates and factors that may contribute to differential responses, highlighting current limitations in linking microbiota composition, microbial function, metabolite production, and clinical outcomes in IBS, and identifying possible directions for future research.

## 1. Introduction

Irritable bowel syndrome (IBS), one of the most prevalent disorders of gut–brain interaction, is characterized by recurrent abdominal pain associated with altered bowel habits and substantial impairment in quality of life (QoL) [1,2]. IBS is diagnosed according to symptom-based Rome criteria, which were developed to enable a positive diagnosis while minimizing the need for extensive diagnostic investigations [3]. The global prevalence of IBS ranges from approximately 13% to 17%, depending on the diagnostic criteria applied, with women and individuals younger than 50 years being disproportionately affected [1,4]. Beyond gastrointestinal symptoms, IBS is frequently associated with extra-intestinal manifestations, including psychological distress, anxiety, and depression [5]. Although its pathogenesis remains incompletely understood, IBS is currently considered a multifactorial disorder resulting from the complex interplay of gut–brain axis dysfunction, autonomic nervous system alterations, environmental and genetic factors, immune activation, increased intestinal permeability, and disturbances in gut microbiota composition and function [5]. Among these mechanisms, the role of the gut microbiota and its metabolic activity has attracted increasing attention, prompting growing interest in microbiota-targeted dietary interventions [2,5]. The gut microbiota comprises a complex ecosystem of bacteria, archaea, fungi, and viruses that establish a dynamic symbiotic relationship with the host and contribute to nutrient metabolism, epithelial barrier integrity, immune regulation, protection against pathogens, and the production of bioactive metabolites [6]. An imbalance in the composition, relative abundance, and functional activity of commensal microorganisms, commonly referred to as dysbiosis, has been increasingly implicated in IBS pathophysiology and may contribute to symptom generation, altered microbial metabolism, and disease heterogeneity [2,5,7].

Although IBS is not traditionally classified as an inflammatory disorder, increasing evidence suggests that a subset of patients exhibit features of low-grade mucosal immune activation. Alterations in mucosal cytokine profiles, together with changes in innate immune signaling pathways, have been reported and may contribute to epithelial barrier dysfunction, altered gut–brain communication, and symptom generation [8,9]. Biomarkers such as fecal calprotectin have also been investigated as indicators of intestinal inflammatory activity, although levels are generally substantially lower than those observed in inflammatory bowel disease [10]. In addition to microbial alterations, several host-related mechanisms are implicated in IBS pathophysiology. Disruption of epithelial barrier integrity may facilitate interactions between luminal factors and the mucosal immune system, while alterations in enterochromaffin cell function and serotonin signaling may contribute to changes in gastrointestinal motility, secretion, and visceral sensation [9]. Furthermore, increased mast cell activation and neuroimmune interactions in close proximity to enteric nerve fibers have been associated with visceral hypersensitivity, a hallmark feature of IBS [8]. Collectively, these findings support the concept that IBS arises from complex interactions among microbial, epithelial, immune, and neural pathways rather than from alterations in gut microbiota alone [8,9].

While the therapeutic landscape for IBS has progressively expanded in parallel with advances in the understanding of its complex pathophysiology, dietary interventions continue to represent some of the most widely adopted strategies for symptom management [11]. Among the available dietary strategies, the low-fermentable oligosaccharides, disaccharides, monosaccharides, and polyols (low-FODMAP) diet has emerged as the most extensively studied intervention and is widely recommended as a first-line approach for symptom management in IBS [2,11]. However, despite its well-established efficacy in improving gastrointestinal symptoms, increasing evidence indicates that FODMAP restriction may alter the microbial and metabolic environment of the gut [2]. In particular, reductions in selected microbial taxa, particularly *Bifidobacterium*, together with changes in the colonic fermentative environment, have raised questions about the longer-term implications of these changes for microbiota-associated metabolic activity and gut homeostasis [12,13,14]. Although *Bifidobacterium* is one of the taxa most consistently affected by both low-FODMAP interventions and prebiotic supplementation, changes in its abundance alone do not necessarily reflect corresponding changes in microbial metabolic activity or clinical outcomes.

In this context, prebiotics, substrates that are selectively utilized by host microorganisms and confer a health benefit, have emerged as microbiota-targeted dietary interventions [15]. Through microbial fermentation, they can promote the growth and activity of specific microbial taxa and enhance the production of microbial-derived metabolites, including short-chain fatty acids (SCFAs), which may contribute to intestinal homeostasis [15,16]. SCFAs have attracted particular interest because of their role in maintaining epithelial barrier integrity, regulating immune responses, suppressing the growth of potentially pathogenic microorganisms, and modulating host metabolism [16]. However, changes in microbial composition or metabolic activity do not necessarily translate into improvements in gastrointestinal symptoms or quality of life [15]. Thus, the potential relevance of prebiotics in IBS may extend beyond symptom relief to their effects on microbial metabolic activity and metabolite production [15,17].

The growing recognition that microbial composition and microbial function do not necessarily overlap has shifted attention from taxonomic changes alone toward the metabolic consequences of microbiota modulation [18,19]. In this context, the effects of prebiotics on microbial-derived metabolites, particularly SCFAs, may provide important information beyond changes in bacterial abundance [19]. This concept may be particularly relevant following a low-FODMAP dietary intervention, where modulation of microbiota-associated metabolic functions could represent an important, yet often overlooked, aspect of dietary management [13,14]. However, whether prebiotic-induced changes in microbial composition are accompanied by measurable changes in microbial metabolic activity and clinically meaningful outcomes remains largely unresolved. Therefore, rather than focusing exclusively on taxonomic changes, increasing attention should be directed toward the capacity of prebiotics to modulate microbial metabolic activity and its potential implications for host health.

This narrative review critically examines the metabolic and clinical implications of dietary prebiotics in IBS, with particular emphasis on their potential role following low-FODMAP restriction and on the rationale for structured reintroduction of tolerated fermentable substrates. We discuss changes in gut microbial composition and function, microbial fermentation and metabolite production, and their potential relationships with gastrointestinal symptoms and other patient-reported outcomes. We also consider factors that may contribute to interindividual variability in responses to specific prebiotic substrates, including baseline microbiota, substrate utilization, dose, and host sensitivity. Finally, we discuss how these considerations may inform precision nutrition approaches aimed at balancing symptom control with the potential metabolic benefits of dietary prebiotics in personalized IBS management. Given the limited availability of studies directly assessing the restoration of microbial metabolic function following low-FODMAP restriction, the review also considers indirect evidence derived from prebiotic interventions in broader IBS populations and from studies examining low-FODMAP reintroduction and personalization strategies.

## 2. Literature Search

A focused literature search was conducted in PubMed/MEDLINE from database inception to September 2026 to identify relevant evidence on dietary prebiotics, low-FODMAP dietary interventions, gut microbiota, microbial metabolism, and clinical outcomes in patients with IBS. Searches were performed using combinations of keywords related to the main concepts addressed in this review, including “irritable bowel syndrome,” “prebiotics,” “low-FODMAP,” “FODMAP reintroduction,” “FODMAP restriction,” “gut microbiota,” “microbial metabolites,” and “short-chain fatty acids.” Additional focused searches were performed to identify studies specifically addressing prebiotic supplementation in IBS and the reintroduction or personalization phases of the low-FODMAP diet, as well as emerging approaches to precision nutrition in this context.

Particular attention was given to randomized controlled trials, prospective intervention studies, systematic reviews and meta-analyses, and relevant mechanistic studies investigating changes in microbial composition or function, microbial fermentation and metabolite production, gastrointestinal symptoms, and patient-reported outcomes. Studies addressing individual responses to specific fermentable or prebiotic substrates following low-FODMAP restriction were also considered. Reference lists of relevant articles and reviews were screened to identify additional studies. Given the narrative nature of the review, the search was intended to identify and critically evaluate relevant evidence rather than to provide an exhaustive or systematic assessment of the literature.

## 3. Low-FODMAP Diets: Clinical Benefits and Microbial and Metabolic Consequences

Food intake is an important trigger of gastrointestinal symptoms in IBS, with up to 50% of patients reporting postprandial exacerbation of symptoms [20]. A considerable proportion of patients with IBS report that food intake triggers or worsens gastrointestinal symptoms, and food-related gastrointestinal complaints are associated with greater symptom severity and impaired quality of life [21]. Among dietary factors, poorly absorbed and rapidly fermentable carbohydrates are particularly relevant, as their ingestion can increase luminal water content and promote microbial fermentation, thereby contributing to intestinal distension and symptom generation in susceptible individuals [22,23]. In a double-blind, placebo-controlled randomized crossover trial, a FODMAP challenge induced a modest increase in IBS symptom severity compared with placebo, whereas gluten did not significantly exacerbate symptoms [24]. These observations provided a rationale for dietary strategies aimed at reducing the intestinal load of fermentable carbohydrates, most notably the low-FODMAP diet [22,25]. FODMAPs comprise fermentable oligosaccharides, disaccharides, monosaccharides, and polyols that are incompletely absorbed in the small intestine and readily metabolized by colonic microorganisms [25]. Their osmotic activity increases luminal water content, whereas microbial fermentation promotes the production of gases and metabolites, including SCFAs [22,25]. While excessive gas production may contribute to abdominal pain, bloating, and altered bowel habits in susceptible individuals, fermentation also generates SCFAs, which are involved in maintaining intestinal homeostasis and regulating host metabolic and immune functions [16,22,25].

FODMAPs are widely distributed throughout the diet, being present in cereals, legumes, selected fruits and vegetables, dairy products, and various sweeteners [25]. Consequently, reducing dietary FODMAPs decreases the availability of fermentable substrates in the intestinal lumen and reduces both the osmotic and fermentative load [2]. This provides a biological rationale for the clinical efficacy of the low-FODMAP diet, while also raising questions about the potential consequences of prolonged restriction. Randomized controlled trials have demonstrated that short-term low-FODMAP interventions can improve global IBS symptoms, including abdominal pain, bloating, and altered bowel habits, compared with control dietary approaches [26,27]. These findings are supported by meta-analytic evidence showing an overall reduction in IBS symptom severity and improvement in quality of life with low-FODMAP interventions [28,29,30]. The available evidence therefore supports the low-FODMAP diet as an effective strategy for short-term symptom management in IBS [2]. However, its clinical use should be considered within a structured dietary approach rather than as prolonged and indiscriminate carbohydrate restriction [31]. Current dietary models generally comprise an initial restriction phase followed by systematic FODMAP reintroduction and subsequent individualization according to symptom tolerance [32].

The clinical benefits of reducing fermentable carbohydrates must be considered alongside the role of these substrates as metabolic inputs for the intestinal microbiota. Diet is a major determinant of gut microbial composition, diversity, and metabolic activity, and changes in the availability of fermentable carbohydrates can therefore modify both microbial ecology and microbiota-associated metabolic functions [12,32]. Consistent with this concept, studies of low-FODMAP interventions have reported changes in specific bacterial populations, particularly reductions in *Bifidobacterium* abundance [12,13,14]. In a randomized controlled trial, a low-FODMAP diet reduced fecal *Bifidobacterium* concentrations despite improving IBS symptoms, providing evidence that clinical symptom improvement and preservation of potentially beneficial microbial populations do not necessarily occur in parallel [13]. A systematic review and meta-analysis of nine randomized controlled trials including 403 patients subsequently confirmed that low-FODMAP interventions consistently reduced *Bifidobacterium* abundance, whereas effects on overall microbial diversity and other specific bacterial taxa were less consistent [14]. These findings indicate that the microbiological consequences of FODMAP restriction are selective rather than necessarily reflecting a generalized loss of microbial diversity.

**Figure 1:**
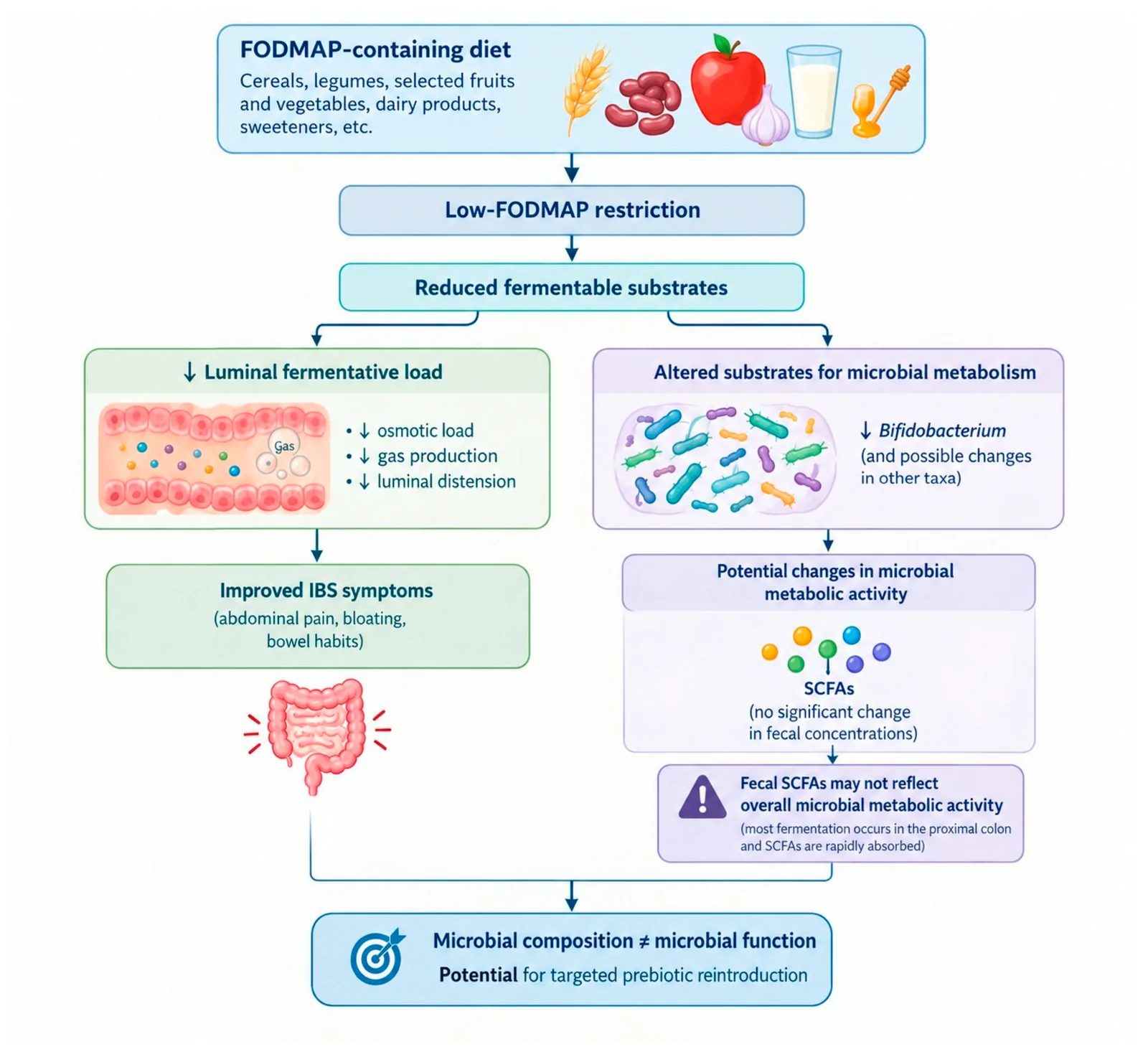
Clinical and microbial consequences of FODMAP restriction in IBS. Low-FODMAP restriction reduces fermentable substrate availability and the luminal fermentative load, improving gastrointestinal symptoms but also modifying gut microbial composition, including a consistent reduction in Bifidobacterium. Despite these changes, fecal SCFA concentrations do not appear to be consistently altered, highlighting the distinction between microbial composition and function and supporting the rationale for targeted reintroduction of tolerated prebiotic substrates. Image generated using ChatGPT (OpenAI, GPT-5.5).

The metabolic consequences of low-FODMAP restriction are more difficult to define. Interestingly, despite the reduced availability of fermentable substrates and associated changes in microbial composition, a systematic review and meta-analysis found no significant differences in fecal SCFA concentrations between low-FODMAP and control dietary interventions [14]. This apparent discrepancy may partly reflect the rapid absorption of SCFAs by colonocytes at their site of production, with only a minor fraction reaching the systemic circulation [33]. Therefore, although low-FODMAP diets consistently alter the abundance of specific microbial taxa, particularly *Bifidobacterium*, these compositional changes do not necessarily translate into measurable alterations in fecal SCFA concentrations [14]. This distinction between microbial composition and function is particularly relevant when considering the metabolic consequences of dietary restriction and is further supported by evidence that low-FODMAP interventions can modify the metabolome independently of taxonomic changes [18] (Figure 1).

## 4. Metabolic Function of Dietary Prebiotics: Potential Relevance in IBS

Since their original definition in the mid-1990s, prebiotics have undergone considerable conceptual refinement. Early definitions described prebiotics as non-digestible food ingredients that resist digestion in the upper gastrointestinal tract, are selectively fermented by intestinal microorganisms, and promote the growth and/or activity of beneficial microbial populations, ultimately improving host health [34]. However, growing knowledge of host–microbiota interactions has broadened this framework, leading the International Scientific Association for Probiotics and Prebiotics to redefine prebiotics as “substrates that are selectively utilized by host microorganisms conferring a health benefit” [17]. Unlike probiotics, which consist of live microorganisms administered to confer a health benefit, prebiotics are dietary substrates that are selectively utilized by resident microorganisms within the gut ecosystem [35]. This concept is particularly relevant in IBS, where alterations in gut microbial composition and function have been implicated in symptom generation and interindividual variability [5,36]. The human gut harbors a highly diverse microbial ecosystem composed of bacteria, archaea, fungi, and viruses that establishes a dynamic relationship with the host and contributes to nutrient metabolism, epithelial barrier integrity, immune regulation, and the production of bioactive metabolites [6]. In contrast, dysbiosis, broadly referring to alterations in microbial composition and/or function, has been associated with gastrointestinal disorders, including IBS [37,38].

Prebiotic substrates can be selectively utilized by specific gut microorganisms and thereby modulate microbial composition, fermentation pathways, and metabolite production [16,17]. Through these mechanisms, the effects attributed to prebiotics extend beyond changes in gut microbial composition and encompass a broad range of physiological processes, including gastrointestinal function, intestinal barrier integrity, immune regulation, and host metabolism [39]. However, the magnitude and nature of these effects may vary according to the type and dose of the prebiotic substrate administered [15]. Importantly, the availability of prebiotic substrates can influence not only the abundance of specific microbial taxa but also their metabolic activity and the profile of metabolites generated through microbial fermentation [16,19,40]. This functional dimension is particularly relevant because microbial metabolites represent an important link between changes in the gut microbiota and host physiology. Among these metabolites, SCFAs are major products of colonic carbohydrate metabolism and primarily comprise acetate, propionate, and butyrate, generally occurring in an approximate ratio of 3:1:1 [41]. Although SCFAs can reach concentrations of nearly 100 mM within the colonic lumen, most are rapidly absorbed by colonocytes, with only a small proportion being excreted in feces [41,42]. These metabolites contribute to gastrointestinal homeostasis through effects on epithelial barrier integrity, immune regulation, host metabolism, and gene expression, including epigenetic regulation [41].

Prebiotics can be classified according to their chemical structure, degree of polymerization, and selective utilization by specific gut microorganisms [39]. Most established prebiotics are carbohydrate-based compounds, including oligosaccharides and non-digestible polysaccharides [16,43]. Among the best-characterized prebiotic substrates are galactooligosaccharides (GOS) and inulin-type fructans (ITF), which have been extensively investigated for their ability to modulate gut microbial composition and activity [39]. More recently, additional substrates such as human milk oligosaccharides, resistant starches, and certain plant-derived compounds have attracted growing interest as potential microbiota-modulating agents, although evidence regarding their clinical relevance in IBS remains limited [39]. In the following sections, the principal classes of prebiotics and their effects on gut microbial composition, microbial metabolism, and host physiological processes are discussed, highlighting mechanisms that may be relevant to IBS (Table 1).

### 4.1. Galactooligosaccharides

GOS are non-digestible galactose-containing oligosaccharides whose structure varies according to their degree of polymerization, branching, and glycosidic linkages [16,44]. Naturally occurring in milk and in several leguminous plant species, GOS are generally classified as α-galacto-oligosaccharides (α-GOS) or β-galacto-oligosaccharides (β-GOS) according to the configuration of their galactosidic bonds [45,46]. While α-GOS are mainly of plant origin, β-GOS, which have been more extensively investigated as prebiotics, are commonly produced through β-galactosidase-catalyzed transgalactosylation of lactose, a well-established enzymatic technology for the commercial production of β-GOS [44,47]. Their physicochemical properties, fermentation characteristics, and biological activities are strongly influenced by their degree of polymerization and glycosidic linkage patterns [46]. GOS are relatively resistant to digestion in the upper gastrointestinal tract, allowing them to reach the colon, where they can be selectively utilized by gut microorganisms [45].

GOS have been investigated for their effects on intestinal health, particularly through their ability to modulate gut microbial composition and activity [48]. They are selectively fermented by gut microorganisms, particularly members of the genus *Bifidobacterium*, and their supplementation has been associated with increased bifidobacterial abundance [49,50,51]. The genus *Bifidobacterium* comprises Gram-positive, anaerobic, saccharolytic commensal bacteria that contribute to intestinal health [52]. Bifidobacterial activity contributes to carbohydrate fermentation and metabolite production, including acetate and other organic acids that may contribute to a lower intestinal pH and inhibit the growth of potentially pathogenic microorganisms [53,54,55]. Moreover, bifidobacteria may exert immunomodulatory effects through the promotion of regulatory pathways, including increased interleukin (IL)-10 production and expansion of regulatory T cells, while attenuating pro-inflammatory responses through reductions in tumor necrosis factor-α (TNF-α), IL-6, IL-8, and IL-1β levels [52,56,57]. GOS have also been reported to improve intestinal barrier integrity in human studies and experimental models, suggesting that their effects on the gut microbiota may have functional consequences for the host beyond changes in bacterial abundance [57,58]. Nevertheless, increases in *Bifidobacterium* abundance alone do not necessarily indicate corresponding changes in microbial metabolic activity, cross-feeding interactions, or clinically meaningful outcomes.

Besides promoting bifidobacterial proliferation, GOS have also been shown to selectively stimulate several *Lactobacillus* species [59]. GOS-promoted colonization by specific *Lactobacillus* strains has been associated with enhanced resistance to pathogen-induced disruption of the intestinal barrier and reduced inflammatory responses, highlighting the broader microbiota-directed effects of GOS beyond bifidobacterial enrichment [59]. Bifidobacteria and lactobacilli are important saccharolytic members of the intestinal microbiota and contribute to microbial fermentation processes [60]. Despite sharing the ability to metabolize carbohydrates, the two genera display distinct metabolic profiles. Lactobacilli primarily produce lactate, whereas bifidobacteria metabolize carbohydrates through the bifid shunt pathway, generating mainly acetate and lactate [61,62]. These differences contribute to the production of fermentation-derived metabolites and broader microbiota-associated metabolic functions.

GOS fermentation can promote metabolic cross-feeding within the gut microbiota, whereby metabolites generated by primary GOS-fermenting bacteria are subsequently utilized by other microbial taxa, contributing to the production of downstream metabolites such as butyrate [63,64]. Thus, the effects of GOS may extend beyond the enrichment of specific bacterial taxa to include changes in microbial metabolic activity and the intestinal metabolite environment [63,65]. This functional dimension is particularly relevant in IBS, where changes in gut microbial composition may be accompanied by alterations in microbial fermentation and metabolite profiles [66,67]. At the same time, the fermentability of GOS may contribute to gastrointestinal symptoms in susceptible individuals, highlighting the need to consider both their microbiota-modulating effects and individual tolerance in IBS [68,69]. Collectively, these microbiological and metabolic effects provide a biological rationale for investigating GOS as a microbiota-targeted intervention in IBS.

### 4.2. Inulin-Type Fructans: Fructooligosaccharides and Inulin

Fructans comprise a structurally diverse family of carbohydrates composed predominantly of fructose units, often linked to a terminal glucose residue [70,71]. They differ in chain length and degree of polymerization, characteristics that influence their physicochemical properties, fermentability, and physiological effects [70]. Inulin-type fructans (ITF) are characterized predominantly by β-(2→1) fructosyl–fructose linkages and include inulin, oligofructose, and short-chain fructooligosaccharides (FOS), which differ primarily in chain length and degree of polymerization [72]. Commercially available ITF may be obtained by extraction from plant sources, particularly chicory root, or through enzymatic synthesis from carbohydrate precursors such as sucrose [72]. Inulin is generally characterized by longer fructan chains, whereas oligofructose and short-chain FOS consist of shorter chains and are generally more rapidly fermented by the gut microbiota [70,71,73]. Owing to the resistance of their β-(2→1) fructosyl–fructose linkages to human digestive enzymes, ITF reach the colon largely intact, where they are metabolized by the gut microbiota and contribute to the production of SCFAs and other microbial-derived metabolites [73,74].

ITF are among the most extensively studied prebiotic substrates and have been associated with bifidogenic effects [74,75]. A systematic review and meta-analysis showed that chicory-derived ITF supplementation at doses of 3–20 g/day significantly increased intestinal *Bifidobacterium* abundance and improved bowel function parameters in healthy individuals [73]. However, the magnitude of the microbiota response may vary according to individual characteristics of the gut microbiota and habitual dietary fiber intake [76]. These findings highlight the importance of the pre-existing microbial and dietary context in determining the response to ITF supplementation.

The metabolic effects of ITF extend beyond the enrichment of bifidobacteria and involve interactions among multiple microbial taxa. Fermentation of oligofructose and inulin can result in the production of acetate and lactate by primary fermenters, which can subsequently serve as substrates for other microorganisms involved in secondary fermentation [77]. Lactate- and acetate-consuming bacteria, including butyrate-producing species such as *Anaerostipes caccae* and *Eubacterium hallii*, can convert these intermediates into butyrate, illustrating how ITF metabolism can promote metabolic cross-feeding within the intestinal ecosystem [77]. Similar interactions have been described between bifidobacteria and *Faecalibacterium prausnitzii*, whereby acetate generated during fructan degradation can support the growth and butyrate production of this butyrate-producing bacterium [78]. Importantly, the extent and direction of these interactions may depend on the ability of individual bacterial strains to degrade fructans, indicating that ITF metabolism is shaped by the functional characteristics of the resident microbial community [78,79].

Importantly, the high fermentability underlying the microbiota-modulating and metabolic effects of fructans may also have clinical implications in IBS. Rapid microbial fermentation of ITF may contribute to the generation of luminal gases and metabolites and, in susceptible individuals, to abdominal distension, bloating, and discomfort [80,81]. However, these symptoms may reflect not only the amount of gas produced but also individual sensitivity to intestinal distension [81]. Conversely, restricting fermentable carbohydrates can reduce gastrointestinal symptoms in IBS but may also reduce the abundance of microbial populations, including bifidobacteria [14,82]. Taken together, these findings support the relevance of fructans as microbiota-targeted prebiotics while highlighting the need to consider their fermentability and individual tolerability in the context of IBS.

### 4.3. Other Prebiotic Classes

Although GOS and ITF represent the most extensively investigated prebiotic substrates, growing attention has been directed toward other compounds with potential prebiotic or microbiota-modulating properties [39]. These include human milk oligosaccharides (HMOs), resistant starches, xylooligosaccharides, polyphenols, and other plant-derived compounds [39,83].

Human milk oligosaccharides (HMOs) are structurally diverse glycans composed of five principal monosaccharide building blocks: glucose, galactose, N-acetylglucosamine, fucose, and sialic acid [83,84]. Most HMOs contain a lactose core at the reducing end and are naturally occurring components of human milk [83]. Traditionally investigated in the context of infant nutrition, HMOs have attracted increasing interest as potential prebiotic substrates beyond infancy because of their ability to selectively modulate the gut microbiota [83,85]. Their microbiota-modulating effects include selectively supporting bifidobacterial growth and altering the abundance and activity of other bacterial taxa, underscoring their potential relevance to intestinal physiology [85,86]. Selected HMOs, including 2′-fucosyllactose (2′FL) and lacto-N-neotetraose (LNnT), can be produced synthetically and have been investigated as orally administered substrates in adults [86,87]. In healthy adults, supplementation with 2′FL and LNnT has been shown to be well tolerated and to increase bifidobacterial abundance [87]. Evidence from patients with IBS indicates that HMO supplementation can modulate the intestinal microbiota. In a randomized, double-blind, placebo-controlled trial including 60 patients with IBS, supplementation with a 4:1 mixture of 2′FL and LNnT increased fecal Bifidobacterium abundance at a dose of 10 g/day and altered overall fecal microbiota composition [88]. However, symptom outcomes did not differ significantly between groups [88]. A subsequent analysis of samples obtained from participants enrolled in the same trial reported changes in fecal and plasma metabolite profiles, as well as alterations in fecal and mucosal microbiota, suggesting that HMO-induced microbiota modulation may extend beyond taxonomic changes to microbial and host metabolic pathways [89]. These findings support the potential relevance of HMOs as microbiota-targeted substrates in IBS, although their clinical and metabolic effects require further investigation.

Resistant starch represents another class of carbohydrate substrates with potential prebiotic properties, particularly because of its capacity to reach the colon and undergo microbial fermentation [90]. Unlike digestible starch fractions, resistant starch escapes digestion and absorption in the small intestine and is fermented by the gut microbiota, contributing to the production of SCFAs and other microbial-derived metabolites [90,91]. Despite its fermentability, resistant starch appears to be generally well tolerated in adults, although gastrointestinal symptoms such as bloating, flatulence, and abdominal discomfort have been reported in some studies [92]. Evidence specifically in IBS remains limited. In a pilot randomized controlled crossover study, supplementation with resistant starch type 2 (RS2) at doses of up to 20 g/day was generally tolerated by patients with IBS, although all tested RS2 doses were associated with increased bloating [93]. These preliminary findings suggest that resistant starch may represent a potentially relevant microbiota-modulating substrate, but its clinical relevance in IBS requires further investigation (Table 1).

**Table 1:** Classification, characteristics, and potential relevance of selected prebiotic substrates in irritable bowel syndrome.

References	Prebiotic Class	Main Structural Characteristics	Principal Microbial Targets	Major Metabolic Effects	Potential Relevance for IBS
[45,46,49,50,51,55,56,57,58,59,63,64,65,68,69]	GOS	Galactose-containing oligosaccharides; α-GOS and β-GOS; β-GOS mainly produced through lactose transgalactosylation	*Bifidobacterium*, *Lactobacillus*	SCFA production, including butyrate through cross-feeding	Microbiota modulation; intestinal barrier support; symptom effect variable
[72,73,74,76,77,78,79,80,81]	ITF	β-(2→1)-linked fructans differing in chain length and degree of polymerization	*Bifidobacterium* spp.; indirect stimulation of butyrate-producing bacteria	SCFA production and cross-feeding	Strong microbiota-modulating activity high fermentability and potential symptom burden
[83,84,85,86,87,88,89]	HMOs	Complex glycans containing glucose, galactose, N-acetylglucosamine, fucose, and sialic acid	Infant-associated bifidobacteria and selected commensals	Bifidogenic activity, modulation of microbial metabolites	Preliminary evidence of microbiota modulation without clear symptom worsening
[90,91,92,93]	Resistant starch	Non-digestible starch fractions resistant to enzymatic digestion in the small intestine (RS1–RS5)	Fermentation by colonic microbiota; cross-feeding	SCFA production, particularly butyrate	Potential metabolic benefits; limited IBS-specific evidence

Abbreviations: GOS: galactooligosaccharides; HMO: human milk oligosaccharide; IBS: irritable bowel syndrome; ITF: inulin-type fructans; SCFA: short-chain fatty acid.

**Figure 2:**
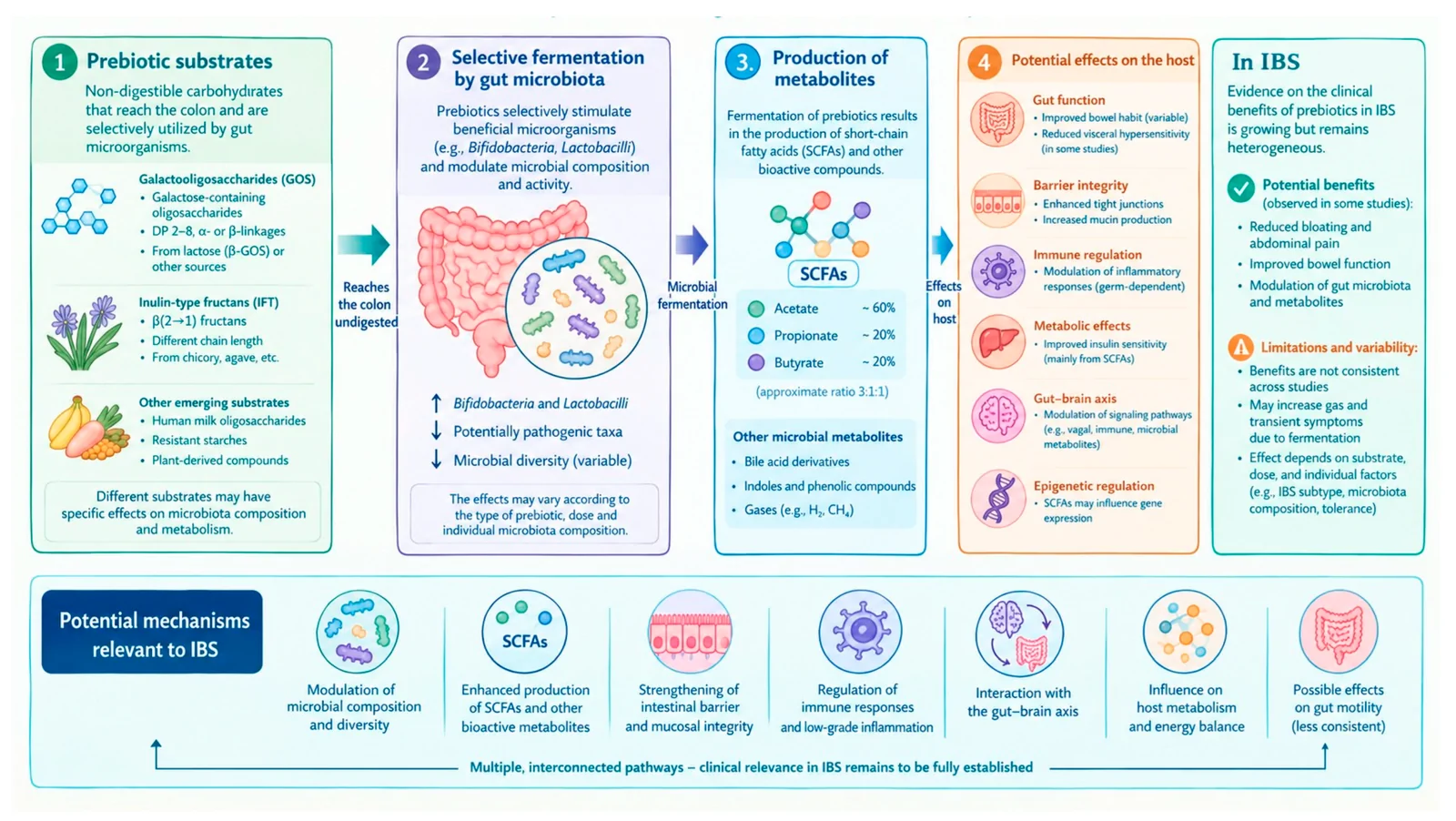
Dietary prebiotics and their potential metabolic effects in irritable bowel syndrome (IBS). Prebiotic substrates, including galactooligosaccharides (GOS), inulin-type fructans (ITF), resistant starches, and human milk oligosaccharides (HMOs), modulate gut microbial composition and activity through selective microbial utilization and fermentation. The resulting metabolites, including short-chain fatty acids (SCFAs), may influence intestinal and host physiology. These effects may vary according to substrate type and dose, gut microbiota composition, and individual symptom susceptibility. Arrows indicate the direction of taxonomic changes most frequently reported in the literature and do not necessarily correspond to metabolic functional changes or clinical benefit. Image generated using ChatGPT (OpenAI, GPT-5.5).

Overall, the available evidence indicates that prebiotic substrates do not exert uniform effects across microbiological and clinical outcomes. A systematic review and meta-analysis of randomized controlled trials in adults with IBS and other functional bowel disorders found that prebiotic supplementation increased the absolute abundance of *Bifidobacterium* but did not consistently improve gastrointestinal symptoms or quality of life [15]. Importantly, symptom responses varied according to prebiotic type and dose, with ITF associated with increased flatulence, whereas GOS showed a different symptom profile, potentially reflecting differences in substrate structure and fermentation characteristics [15]. The structural characteristics of α-GOS and β-GOS may also influence their microbial selectivity and fermentation patterns, with β-GOS generally showing greater bifidogenic selectivity than plant-derived α-GOS [47]. These findings underscore the substrate-specific nature of prebiotic effects and support consideration of substrate type, dose, fermentability, and individual tolerance when evaluating their potential role in IBS (Table 2, Figure 2).

## 5. Low-FODMAP Diets and Prebiotic Substrates: Balancing Symptom Control and Microbial Function

### 5.1. The Fermentation Paradox

As discussed in the previous sections, prebiotics can modulate gut microbial activity, with potential health effects mediated, at least in part, through selective microbial utilization, fermentation, and SCFA production [19,94]. Owing to their structural characteristics, many prebiotic substrates resist digestion in the upper gastrointestinal tract and reach the large intestine, where they can serve as substrates for resident gut microorganisms, particularly *Bifidobacterium* species [94]. SCFAs contribute to shaping the gut environment and influence intestinal barrier function, immune regulation, epithelial metabolism, and broader host physiological processes, providing a biological basis for the potential health effects associated with microbial fermentation of prebiotic substrates [19,41]. Importantly, SCFA production is also influenced by bacterial cross-feeding, whereby metabolites generated by primary fermenters are subsequently utilized by other microbial taxa, contributing to the overall balance of SCFA production and the efficient utilization of substrates reaching the colon [41]. From this perspective, microbial fermentation is an essential component of the metabolic activity associated with prebiotic substrates and cannot be considered merely a secondary consequence of their consumption.

In IBS, the physiological consequences of microbial fermentation occur within a complex pathophysiological context involving alterations in gut microbial composition and function, intestinal barrier integrity, visceral sensitivity, and gut–brain axis signaling [7,95]. These mechanisms may interact with one another and contribute to the variable clinical response to fermentable dietary substrates observed among patients with IBS [96,97]. In particular, altered intestinal barrier integrity and visceral hypersensitivity may increase the response to luminal stimuli, while alterations in gut microbial composition and function may influence intestinal physiology and microbial metabolite profiles [95,98,99].

Within this complex pathophysiological framework, dietary factors may also influence gastrointestinal symptoms, particularly through the fermentation of poorly absorbed carbohydrates [95]. Upon ingestion of these substances, patients with IBS may experience increased abdominal symptoms, including discomfort and pain, compared with healthy individuals [81]. Importantly, patients with IBS may report more carbohydrate-related symptoms than controls despite no significant differences in gas production, consistent with increased sensitivity to intestinal distension, whereby a physiological increase in gas and luminal volume may be perceived as symptomatic in susceptible individuals [81]. The complexity of this relationship is further illustrated by a randomized crossover pilot study in patients with IBS, in which α-galactosidase administered with oligosaccharide-rich meals did not significantly reduce gastrointestinal symptoms or breath hydrogen and methane compared with placebo [100]. These findings, together with evidence of heightened sensitivity to intestinal distension, suggest that the relationship between carbohydrate fermentation, gas production, and symptom perception is not straightforward and may involve factors beyond the absolute amount of intestinal gas produced [81,100]. Taken together, the gastrointestinal response to fermentable carbohydrates varies substantially among individuals with IBS, both in the type and severity of symptoms experienced, and is unlikely to be determined solely by the extent of fermentation or gas production.

This creates a functional paradox in the dietary use of prebiotic substrates in IBS. While their fermentability underlies many of their microbiota-mediated metabolic effects, extensive fermentation may compromise gastrointestinal tolerance in susceptible individuals. The clinical relevance of this balance is illustrated by studies of different prebiotic substrates. ITF are consistently associated with bifidogenic effects and enhanced microbial fermentation, but their rapid fermentation may also increase flatulence and other gastrointestinal symptoms in some individuals with IBS [73,74,80]. Similarly, GOS can selectively stimulate bifidobacteria and increase microbial fermentation, while their fermentability may increase bloating and abdominal symptoms in individuals with limited tolerance [49,68,69]. Resistant starch provides another example: in addition to SCFAs, its fermentation generates gases such as hydrogen, methane, and carbon dioxide, whose accumulation and release into the intestinal lumen may contribute to flatulence and discomfort, particularly in individuals with IBS [91,93]. However, the effects of fermentation are unlikely to depend solely on whether a substrate is classified as a prebiotic. Rather, they may be influenced by the type and physicochemical characteristics of the substrate, which in turn affect the rate and extent of fermentation, the dose administered, and the composition and metabolic capacity of the resident microbiota [40,76,101,102]. Habitual dietary context may also contribute to this variability, as an intervention study in healthy adults showed that habitual dietary fiber intake influenced the gut microbiota response to an inulin-type fructan prebiotic [76]. These factors may contribute to differences in the gastrointestinal consequences of fermentation and help explain why the same prebiotic substrate can produce different clinical responses among individuals with IBS.

Accordingly, fermentability should not be regarded as inherently beneficial or detrimental, but as a functional property whose consequences depend on the interaction between the dietary substrate, the intestinal microbial ecosystem, and the host. This duality is particularly relevant in IBS, where the metabolic benefits associated with microbial fermentation must be considered in relation to its potential to generate symptoms.

### 5.2. Low-FODMAP Restriction and Loss of Fermentable Substrates

The fermentation paradox described above is particularly relevant to the low-FODMAP diet, which restricts the intake of poorly absorbed and highly fermentable carbohydrates to alleviate gastrointestinal symptoms [28,29,30]. Restriction of fermentable carbohydrates can reduce the osmotic and fermentative load in the distal small intestine and proximal colon and consequently improve gastrointestinal symptoms in patients with IBS [25,103]. However, reducing the intake of fermentable substrates also decreases the availability of carbohydrates that can be utilized by the gut microbiota [14]. Low-FODMAP interventions have been associated with a reduced abundance of *Bifidobacterium*, whereas consistent effects on overall microbial diversity or on the abundance of other specific taxa have not been demonstrated [14]. Similarly, available evidence has not demonstrated consistent changes in fecal SCFA concentrations following low-FODMAP interventions [14]. Nonetheless, as more than 95% of SCFAs produced in the large-bowel lumen are absorbed, fecal SCFA concentrations provide only a limited indication of colonic SCFA production, and fecal or luminal concentrations cannot be used as reliable proxies for production rates [42,104]. Future studies should consider combining fecal SCFA measurements with complementary assessments of microbial function, including metabolomic profiling, circulating microbial metabolites, breath gas measurements, and functional microbiome analyses. Although these approaches do not directly quantify colonic SCFA production rates, they can provide indirect and complementary insights into microbial activity, metabolic outputs, and host–microbiota interactions. As such, each captures distinct aspects of microbial function rather than SCFA production per se. Therefore, integrated assessment strategies may offer a more comprehensive characterization of microbial metabolic function than fecal metabolites alone.

Therefore, symptomatic improvement following dietary restriction does not necessarily imply parallel improvements in microbial composition or function. This is particularly relevant because several FODMAPs, including fructans and GOS, are also fermentable substrates with prebiotic properties [7,19]. Their restriction may therefore reduce not only the fermentative stimuli associated with symptom generation but also exposure to substrates that support microbial growth, fermentation, and the production of microbial-derived metabolites [42,105]. Experimental evidence indicates that reducing dietary FODMAP intake can alter the colonic luminal environment, including microbial composition and fermentation-related measures [12]. Conversely, the addition of a prebiotic substrate such as β-GOS during a low-FODMAP intervention has been investigated as a strategy to maintain prebiotic substrate availability during dietary restriction [106]. A dose of 1.4 g/day increased the proportion of patients reporting adequate symptom relief compared with a sham diet and placebo [106]. However, β-GOS supplementation did not prevent the reduction in fecal *Bifidobacterium* associated with the low-FODMAP diet, and fecal butyrate concentrations remained lower than in the control group [106]. Thus, maintaining exposure to a prebiotic substrate during carbohydrate restriction did not fully preserve the microbial and metabolic features affected by the low-FODMAP intervention [106]. These findings illustrate the complexity of manipulating fermentable substrates in IBS: the addition of a prebiotic substrate may influence clinical symptoms without necessarily reversing all microbiota-related consequences of carbohydrate restriction. However, the interpretation of microbiological changes following a low-FODMAP intervention is further complicated by the absence of a universally accepted marker of microbial metabolic function. Depending on the study, this concept has been inferred from microbial composition, fecal SCFA concentrations, broader metabolite profiles, or other indicators of microbial activity, although these measures are not necessarily interchangeable and may not evolve in parallel. Consequently, support of microbial metabolic function should not be interpreted exclusively as a return to pre-restriction values, convergence toward a control-diet profile, or improvement in clinical symptoms.

Accordingly, the microbiological effects of a low-FODMAP diet should be interpreted in the context of its primary clinical objective: symptom control. The reduction in fermentable substrates may be beneficial for patients whose symptoms are driven or amplified by fermentation-related luminal stimuli, but prolonged or unnecessarily restrictive intake may also reduce exposure to substrates with potential microbiota-mediated metabolic effects. This balance provides the rationale for moving beyond prolonged blanket restriction toward the structured reintroduction of tolerated fermentable substrates.

### 5.3. Prebiotics Within a Personalized Low-FODMAP Strategy

The reintroduction phase therefore represents an important component of the low-FODMAP approach. Rather than maintaining broad and prolonged restriction, systematic reintroduction allows individual FODMAP triggers and tolerated amounts to be identified, potentially increasing dietary variety while maintaining symptom control [31]. Beyond its role in dietary management, the reintroduction of fermentable carbohydrates may also have relevance for the intestinal microbiota and microbial metabolism. From a prebiotic perspective, reintroduction can restore exposure to substrates that support microbial growth, fermentation, cross-feeding interactions, and the production of microbial-derived metabolites [16,41,77,78]. Fructans and GOS, in particular, are fermentable carbohydrates with established prebiotic properties [45,72] and are among the substrates whose intake may be reduced during a low-FODMAP restriction phase [12]. The potential benefits of reintroduction are also supported by clinical evidence. In a randomized controlled trial of patients with IBS, symptom improvement achieved during the initial low-FODMAP phase was maintained after the reintroduction of selected FODMAP-containing foods, while fiber intake increased following reintroduction [107]. These findings suggest that dietary restriction can subsequently be relaxed without necessarily compromising symptom control, while potentially improving dietary variety and fiber intake [107].

Despite its clinical importance, the reintroduction phase has been less extensively studied than the initial restriction phase, and evidence to guide its implementation remains limited [31]. Reintroduction generally involves staged and dose-specific challenges designed to assess tolerance, with the longer-term aim of improving dietary variety and nutritional adequacy [31]. The sequence, dose, and frequency of individual challenges may need to be adapted according to symptom responses rather than following a uniformly fixed schedule [31]. This is particularly relevant because responses to individual FODMAP groups can vary substantially among patients with IBS. In a blinded reintroduction study of 117 patients with IBS who had responded to a low-FODMAP diet, 85% of FODMAP challenges triggered symptom recurrence, but the number and type of triggers varied between individuals [108]. Fructans and mannitol were the most frequent triggers, while GOS triggered symptoms in approximately one-third of participants [108]. Similarly, a randomized reintroduction trial showed that fructans and GOS were associated with increased abdominal pain, while GOS also increased bloating in patients who had previously responded to FODMAP restriction [109]. These findings indicate that fermentable substrates should not necessarily be considered a homogeneous dietary category, as their tolerability may differ substantially among individuals and between specific FODMAP groups.

Accordingly, reintroduction should be considered an individualized process aimed at identifying the types and amounts of fermentable carbohydrates that can be tolerated while maintaining symptom control and dietary variety [31]. Interindividual variability in response to dietary interventions may also be influenced by factors beyond microbial composition alone. Potential contributors include IBS subtype, habitual dietary patterns, baseline microbiota characteristics, including the abundance of key taxa such as *Bifidobacterium*, concomitant medications or probiotic use, and recent antibiotic exposure. These variables may influence both microbial and clinical responses to fermentable substrates and prebiotic interventions and may partly contribute to the heterogeneity observed across studies. However, they have not been consistently assessed or controlled for in the available literature. Implementation under the guidance of a dietitian with specific expertise in gastrointestinal disorders is recommended, particularly when multiple dietary restrictions or complex symptom patterns are present [31].

Importantly, the potential microbiological implications of this approach extend beyond the short-term reintroduction phase. In a long-term follow-up study of patients who underwent structured restriction, reintroduction, and personalization, *Bifidobacterium* abundance after 12 months was not significantly different from baseline values [110]. However, the study included a limited number of participants at follow-up and lacked a concurrent control group, precluding conclusions regarding microbiota restoration or equivalence to baseline conditions. Furthermore, total fecal SCFA concentrations, including acetate, propionate, and butyrate, remained lower than baseline values [110]. These findings suggest that the persistence or recovery of individual microbial taxa does not necessarily coincide with corresponding changes in fecal microbial metabolite profiles.

Changes in the abundance of individual microbial taxa, such as *Bifidobacterium*, do not necessarily imply corresponding changes in microbiota-associated metabolic outputs or clinical outcomes. This observation reinforces the concept that taxonomic and functional outcomes may not fully overlap and suggests that assessment of microbial metabolites may provide complementary information beyond measures of microbial composition alone. Accordingly, dietary fibers and fermentable substrates should be considered in terms of their specific functional properties rather than viewed as a homogeneous group. Different fibers can elicit distinct physiological and microbiological responses, suggesting that their use in IBS should take into account fermentation characteristics, gastrointestinal effects, and individual tolerance rather than relying on a uniform recommendation [111]. In this context, selected prebiotic substrates may represent potential components of a personalized dietary strategy when they can be incorporated at individually tolerated doses while providing substrates for microbial activity [111].

**Figure 3:**
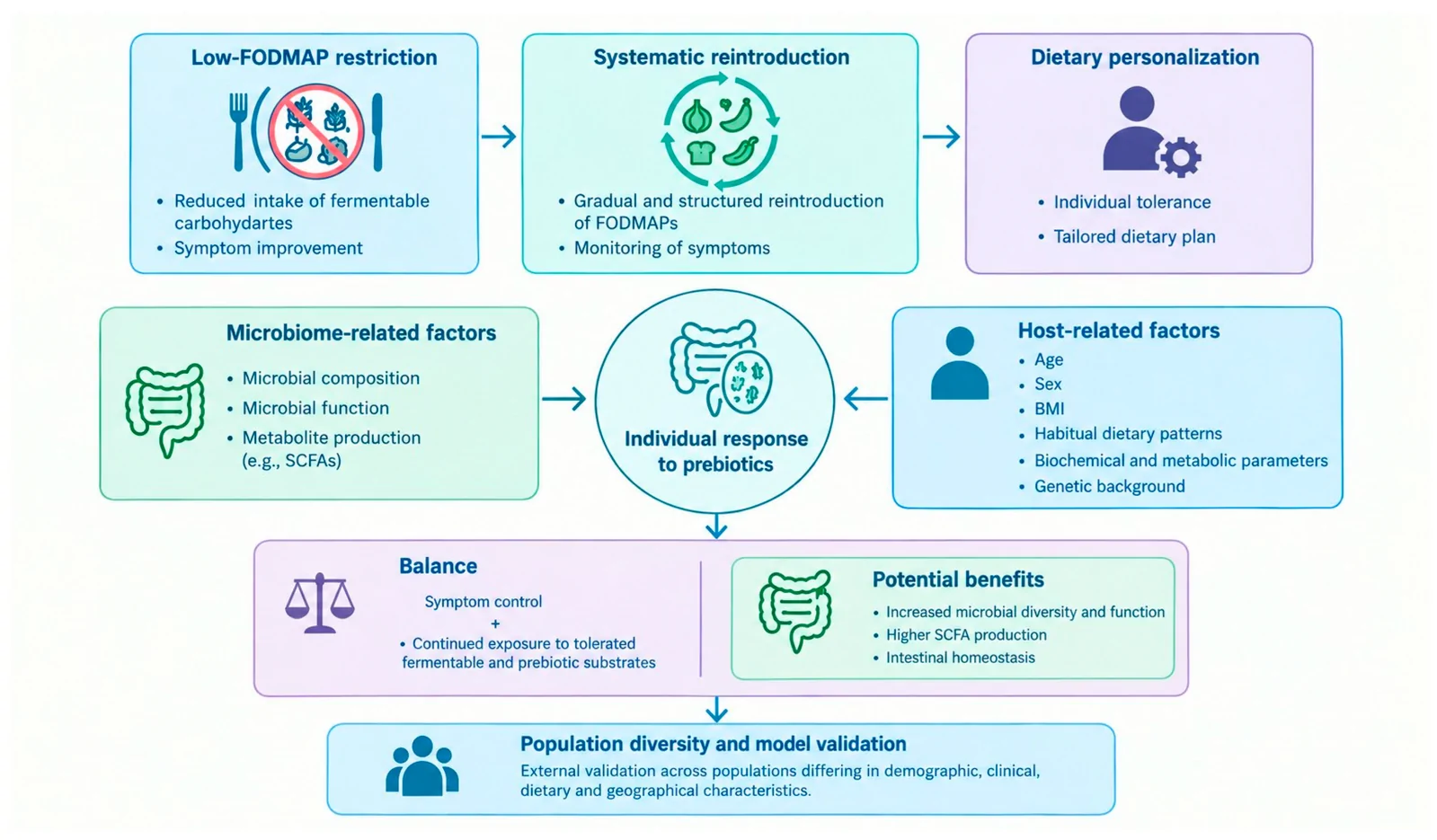
Conceptual framework illustrating the transition from low-FODMAP restriction to systematic reintroduction and dietary personalization in IBS. The framework highlights the balance between symptom control and continued exposure to tolerated fermentable and prebiotic substrates, considering the contribution of microbiome-related and host-related factors to individual dietary responses. It also highlights the potential role of artificial intelligence in integrating individual-level data and the need for external validation of predictive models across diverse populations. Abbreviations: FODMAP: fermentable oligosaccharides, disaccharides, monosaccharides and polyols; SCFA: short-chain fatty acid. Image generated using ChatGPT (OpenAI, GPT-5.5).

Overall, the available evidence supports a shift from considering fermentable carbohydrates simply as dietary components to be restricted or avoided toward a more individualized approach that considers their potential benefits and limitations. The objective of dietary management may therefore be to identify an appropriate balance between symptom control and continued exposure to tolerated fermentable and prebiotic substrates. Rather than viewing fermentable carbohydrates solely as symptom triggers, low-FODMAP strategies should also consider their potential role as substrates supporting microbiota-associated metabolic functions and intestinal homeostasis (Figure 3). Importantly, tolerability may be dose-dependent, and symptom worsening at higher doses does not necessarily preclude the use of lower amounts that remain clinically tolerable while still contributing to microbial substrate availability. This principle may be particularly relevant when incorporating prebiotic-containing foods into individualized reintroduction protocols [15].

## 6. Conclusions and Future Opportunities

Dietary prebiotics represent an important interface between diet, the gut microbiota, and host metabolism, with potential relevance to the pathophysiology and management of IBS. Their selective utilization by gut microorganisms can influence microbial composition and activity, promote fermentation and cross-feeding, and contribute to the production of SCFAs and other microbial metabolites that may affect intestinal barrier function, immune responses, and host physiology. However, the clinical relevance of these metabolic effects in IBS remains incompletely defined, and available evidence indicates substantial heterogeneity according to the type of prebiotic substrate, dose, microbiota composition, and individual tolerance.

The low-FODMAP diet illustrates the complexity of this relationship. Restriction of poorly absorbed and highly fermentable carbohydrates can provide clinically meaningful short-term symptom relief, but it may also reduce exposure to fermentable substrates with prebiotic properties and alter selected components of the gut microbiota. Importantly, changes in microbial composition do not necessarily parallel changes in microbial metabolic activity, and conventional fecal measurements may not fully capture these functional effects. Thus, symptom improvement should not necessarily be interpreted as evidence of parallel improvements in all microbiological or metabolic outcomes.

These observations highlight the need to distinguish between symptom-oriented outcomes and microbiota-associated functional outcomes when evaluating dietary interventions in IBS. Moreover, no universally accepted marker of microbial metabolic function currently exists. Measures such as microbial composition, SCFA concentrations, metabolomic profiles, and clinical outcomes capture different dimensions of the host–microbiota interaction and should not be considered equivalent indicators of functional recovery or support. In this context, prebiotics should not be considered uniformly beneficial or detrimental in IBS. Fermentable substrates may support microbial metabolism and contribute to intestinal homeostasis, whereas excessive or poorly tolerated fermentation may exacerbate gastrointestinal symptoms in susceptible individuals. The available evidence therefore supports an individualized approach in which the low-FODMAP diet is used primarily as a short-term symptom-management strategy, followed by systematic reintroduction and personalization of tolerated fermentable carbohydrates. Such an approach may help maintain dietary variety and exposure to selected prebiotic substrates while minimizing symptom generation. Within this framework, precision nutrition may provide an opportunity to better account for marked interindividual variability and develop more tailored dietary strategies that consider nutritional, genomic, epigenetic, microbiome, and environmental factors [112,113].

In addition to microbiome-related features, individual responses to dietary interventions may be influenced by host-related factors, including age, sex, body mass index, habitual dietary patterns, biochemical and metabolic characteristics, and genetic background. Emerging approaches, including artificial intelligence, may contribute to the development of individualized dietary interventions by integrating these complex biological and individual-level data. However, important challenges remain, including multi-omics data integration and standardization, limited model generalizability across populations, algorithmic opacity, data privacy and security concerns, ethical considerations, and robust validation before implementation in clinical practice [114,115]. Predictive models developed in specific cohorts may not perform similarly across populations differing in demographic characteristics, dietary habits, geographic background, or clinical characteristics, highlighting the need for external validation in diverse populations. Other emerging approaches, including sensory analysis and wearable monitoring, may also contribute to the broader framework of personalized nutrition [116].

Several important knowledge gaps remain. Available studies vary widely in IBS phenotype, prebiotic type, dosage, intervention duration, and outcome assessment [15]. Moreover, relatively few studies have simultaneously evaluated microbial composition, microbial function, metabolite production, and clinical outcomes, limiting mechanistic interpretation of observed findings [5,66]. Future research should prioritize standardized, multidisciplinary study designs integrating clinical, microbiological, and metabolomic endpoints. In parallel, dietary strategies may benefit from moving beyond taxonomic restoration alone and toward the preservation or recovery of microbial function, recognizing that compositional and functional responses do not necessarily evolve in parallel [5,66]. Furthermore, the identification of clinically meaningful functional biomarkers remains a priority, as changes in microbial composition alone may not adequately reflect alterations in microbial metabolic activity.

Further experimental studies using both established and emerging approaches are needed to determine which prebiotic substrates, doses, and combinations may provide clinically relevant benefits in specific IBS phenotypes and to clarify the relationships among microbial composition, microbial function, metabolite production, and symptom responses. Integrating microbiota- and metabolite-based outcomes with clinical and dietary measures may help move the field from broad dietary restriction toward more targeted and personalized nutritional strategies for IBS, while improving our ability to preserve and support microbiota-associated metabolic functions following low-FODMAP intervention.

## Figures and Tables

**Table 2:** Summary of human studies evaluating the effects of prebiotics on clinical outcomes and gut microbiota composition in healthy individuals and patients with irritable bowel syndrome.

Reference	Prebiotic Class	Population	Country	Study Design	Intervention	Main Microbiological/Metabolic Findings	Main Clinical Findings
Systematic reviews and meta-analyses							
Wilson et al., 2019 [15]	ITF, GOS, partially hydrolyzed guar gum, and pectin	Adults with IBS or other functional bowel disorders (11 RCTs, *n* = 729)	Multiple countries	Systematic review and meta-analysis of randomized controlled trials	Prebiotic supplementation versus placebo across 11 RCTs	Increased fecal *Bifidobacterium* abundance; no significant effects on *Lactobacillus* abundance.	No overall improvement in symptoms or quality of life; improved flatulence with non-ITF prebiotics and doses ≤ 6 g/day; worsened flatulence with ITF.
Nagy et al. [73]	ITF	Healthy individuals and populations with health impairments, including gastrointestinal disorders (50 studies, 2525 participants)	Multiple countries	Systematic review and meta-analysis of randomized controlled trials	Chicory-derived ITF (3–20 g/day; ≥7 days)	Increased *Bifidobacterium* abundance; significant bifidogenic effects in healthy individuals and most clinical populations, but not in gastrointestinal disorders.	Increased stool frequency and improved bowel function parameters in healthy subjects; no consistent benefits in gastrointestinal disorders; mild abdominal symptoms, particularly flatulence.).
Sobh et al., 2022 [92]	Resistant starch	2263 participants from 39 trials; healthy individuals and subjects with medical conditions	Multiple countries	Systematic review of randomized controlled trials	Resistant starch supplementation (6.6–60 g/day; 2 days–24 weeks), predominantly RS2 at 20–40 g/day	Increased SCFA production in most studies (70%)	Good tolerability; occasional flatulence, bloating, abdominal discomfort and increased stool frequency; rare treatment discontinuation.
Prebiotic supplementation studies							
Depeint et al., 2008 [49]	GOS	Healthy volunteers (*n* = 59)	United Kingdom	Randomized, double-blind, crossover, placebo-controlled intervention study	β-GOS (3.6 or 7 g/day) versus placebo (sucrose) and commercial GOS (V-GOS) for 7-day treatment periods with 7-day washouts	Dose-dependent increase in fecal Bifidobacterium; greater bifidogenic effect of β-GOS than commercial GOS.	Good tolerability at 3.6 g/day; abdominal discomfort and diarrhea in two participants at 7 g/day.
Davis et al., 2011 [50]	GOS	Healthy volunteers (*n* = 18)	United States	Single-arm dose-escalation intervention study	GOS-containing confectionery; sequential doses of 0, 2.5, 5 and 10 g/day, each administered for 3 weeks following a 2-week baseline period, with a final 2-week washout	Dose-dependent increase in Bifidobacterium/Bifidobacteriaceae; reduction in Bacteroides/Bacteroidaceae.	No clinical outcomes or gastrointestinal symptom outcomes assessed
Teuri et al., 1998 [68]	GOS	Healthy volunteers (*n* = 12)	Finland	Single-arm intervention study	Yogurt containing 15 g/day GOS for 2 weeks	No significant change in fecal Bifidobacterium; increased MRS-growing bacterial populations.	Increased defecation frequency; increased flatulence and gastrointestinal symptoms.
Li et al., 2025 [70]	ITF	131 adults (healthy normal-weight and overweight/obese participants); inulin *n* = 44, FOS *n* = 43, placebo *n* = 44	China	Randomized, double-blind, placebo-controlled clinical trial	15 g/day inulin or 15 g/day FOS for 4 weeks versus maltodextrin placebo	Increased *Lactobacillus*; reduced *Ruminococcus*, Firmicutes, and Clostridia; association between reduced *Ruminococcus* abundance and improved glycemic outcomes	Improved glycemic control and reduced homocysteine with inulin in overweight/obese participants; no serious adverse events.
Elison et al., 2016 [87]	HMOs	Healthy adult volunteers (*n* = 100)	Denmark	Randomized, double-blind, placebo-controlled, parallel-group dose-finding trial	2′FL, LNnT, or 2′FL:LNnT mixture (2:1), 5, 10 or 20 g/day for 2 weeks; glucose placebo	Increased *Bifidobacterium* and Actinobacteria; reduced Firmicutes and Proteobacteria; no significant changes in fecal SCFA.	Good tolerability and safety; mild flatulence, bloating and loose stools at higher doses; minor changes in stool frequency and consistency.
Iribarren et al., 2020 [88]	HMOs	IBS patients (*n* = 60; IBS-C, IBS-D and IBS-M)	Sweden	Randomized, double-blind, placebo-controlled, parallel-group trial	2′FL + LNnT (4:1 mixture), 5 or 10 g/day for 4 weeks; glucose placebo	Increased fecal *Bifidobacterium* abundance with 10 g/day; modulation of overall microbiota composition; increased Actinobacteria, *Alistipes*, Bacteroides and Prevotella.	No symptom deterioration; no worsening of IBS severity, bowel habits, anxiety or depression; improved gastrointestinal symptoms at follow-up in the 10 g group.
Iribarren et al., 2021 [89]	HMOs	IBS patients (*n* = 58; IBS-C, IBS-D, IBS-M) with moderate-to-severe symptoms	Sweden	Secondary analysis of participants from the same trial [88]	2′FL/LNnT (4:1 mixture of 2′FL and LNnT), 5 or 10 g/day for 4 weeks; glucose placebo	Increased fecal and mucosal *Bifidobacterium* (*B. adolescentis*, *B. longum*); modulation of microbiota and metabolite profiles; no significant changes in mucosal host response.	No worsening of IBS severity or psychological symptoms; study not powered to assess symptom improvement.
So et al., 2022 [93]	Resistant starch (RS2)	IBS patients (*n* = 6) and healthy controls (*n* = 5)	Australia	Pilot randomized, single-blind, crossover trial	RS2 (0–20 g/day), sugarcane bagasse (5–15 g/day), or their combination (20–30 g/day total fiber), escalating doses over 9-day intervention periods in a crossover design	Not assessed	No significant change in overall gastrointestinal symptoms; increased bloating with RS2 and higher fiber doses; generally good tolerability of RS2 up to 20 g/day.
Low-FODMAP restriction studies							
Staudacher et al., 2012 [82]	GOS/FOS	IBS patients with bloating and/or diarrhea (*n* = 41)	United Kingdom	Randomized controlled trial (parallel-group, 4-week intervention)	Low-FODMAP diet (reduced dietary intake of FOS and GOS for 4 weeks)	Reduced fecal *Bifidobacterium* concentration and relative abundance; no significant changes in total bacterial counts, *Faecalibacterium prausnitzii*, Bacteroides-*Prevotella*, SCFA concentrations or fecal pH	Improved symptom control; reduced bloating, abdominal pain, flatulence and overall symptoms; reduced stool frequency; increased normal stool consistency.
FODMAP rechallenge/reintroduction studies							
Shepherd et al., 2008 [80]	ITF	IBS patients with fructose malabsorption who had previously responded to a low-FODMAP diet (*n* = 25)	Australia	Randomized, double-blind, quadruple-arm, placebo-controlled crossover rechallenge trial	Graded fructan, fructose, fructan + fructose, or glucose challenges during controlled low-FODMAP feeding; maximum 2-week intervention periods with washout phases	Not assessed	Inadequately controlled symptoms in 77% during fructan challenge; increased overall symptoms, bloating, abdominal pain and flatulence; dose-dependent symptom induction.
Major et al., 2017 [81]	ITF	IBS patients with bloating (*n* = 29) and healthy controls (*n* = 29)	United Kingdom	Randomized, double-blind, 3-period crossover trial with parallel healthy-control cohort	Single oral challenge with 40 g inulin, fructose, or glucose on separate study days	Increased breath hydrogen, colonic gas and colonic volume; correlation between symptom intensity and colonic gas accumulation.	Increased symptom response, bloating, abdominal pain/discomfort and flatulence after inulin; symptom threshold reached by 45% of IBS patients.

Abbreviations: 2′FL: 2′-fucosyllactose; FODMAP: fermentable oligosaccharides, disaccharides, monosaccharides and polyols; FOS: fructooligosaccharides; GOS; galactooligosaccharides; HMO: human milk oligosaccharide; IBS: irritable bowel syndrome; IBS-C: irritable bowel syndrome with constipation; IBS-D: irritable bowel syndrome with diarrhea; IBS-M: irritable bowel syndrome with mixed bowel habits; ITF: inulin-type fructans; LNnT: lacto-N-neotetraose; RS2: resistant starch type 2; RCT: randomized controlled trial; SCFA: short-chain fatty acid.

## Data Availability

No new data were created or analyzed in this study. Data sharing is not applicable to this article.

## Abbreviations

The following abbreviations are used in this manuscript:

FOS	Fructooligosaccharides
GOS	Galactooligosaccharides
HMO	Human milk oligosaccharides
IBS	Irritable bowel syndrome
ITF	Inulin-type fructans
SCFA	Short-chain fatty acid
